# Four calcium signaling pathway-related genes were upregulated in microcystic adnexal carcinoma: transcriptome analysis and immunohistochemical validation

**DOI:** 10.1186/s12957-022-02601-6

**Published:** 2022-05-04

**Authors:** Shuaixia Yu, Yang Wang, Baijie Tang, Xiang Liu, Linhong Song, Gang Xu, Hong Zhu, Huajun Sun

**Affiliations:** 1 Department of Pathology, Sichuan Academy of Medical Sciences & Sichuan Provincial People’s Hospital, Chinese Academy of Sciences Sichuan Translational Medicine Research Hospital, No. 32, West Second Section, First Ring Road, Chengdu, 610072 China; 2 Department of Pathology, Sichuan Provincial People’s Hospital, University of Electronic Science and Technology of China, Chengdu, 611731 China; 3 Department of Pathology, Shenzhen People’s Hospital, The Second Clinical Medical College, Jinan University, The First Affiliated Hospital, Southern University of Science and Technology, Shenzhen, 518020 Guangdong China

**Keywords:** Microcystic adnexal carcinoma, Molecular markers, Transcriptome, Calcium signaling pathway, Diagnostic markers

## Abstract

**Background:**

Microcystic adnexal carcinoma (MAC) is a skin cancer with challenges in diagnosis and management. This study was aimed to detect molecular alterations of MAC and guide its pathologic diagnosis and treatment.

**Methods:**

We performed transcriptome analysis on 5 MAC and 5 normal skin tissues, identified the differentially expressed genes, and verified them by immunohistochemistry.

**Results:**

Three hundred four differentially expressed genes (DEGs) in MAC were identified by next-generation transcriptome sequencing, among which 225 genes were upregulated and 79 genes were downregulated. Four genes of the calcium signaling pathway, including *calcium voltage-gated channel subunit alpha 1 S* (*CACNA1S*), *ATPase sarcoplasmic/endoplasmic reticulum Ca2+ transporting 1* (*ATP2A1*), *ryanodine receptor 1* (*RYR1*), and *myosin light chain kinase 3* (*MYLK3*), were upregulated and then been verified by immunohistochemistry. The expression of CACNA1S, ATP2A1, RYR1, and MYLK3 was upregulated in MAC compared with normal sweat glands and syringoma tumor cells and was generally negative in trichoepithelioma and infundibulocystic type basal cell carcinoma.

**Conclusions:**

The four genes of the calcium signaling pathway were upregulated in MAC at both RNA and protein levels. *CACNA1S*, *ATP2A1*, *RYR1*, and *MYLK3* may be new diagnostic molecular markers and therapeutic targets for MAC.

**Supplementary Information:**

The online version contains supplementary material available at 10.1186/s12957-022-02601-6.

## Key messages

1. Microcystic adnexal carcinoma (MAC) is a rare, well-differentiated cutaneous adnexal carcinoma, with challenges in diagnosis and management. There have been no transcriptomic analyses of MAC.
2. We aimed to detect molecular alterations of MAC and found that the four genes (*CACNA1S*, *MYLK3*, *RYR1*, and *ATP2A1*) of the calcium signaling pathway were upregulated in the RNA level in MAC.
3. Meanwhile, we verified the protein expression of CACNA1S, MYLK3, RYR1, and ATP2A1 in MAC, normal sweat glands, and histologic mimics of MAC, and we found that the four genes may be useful diagnostic markers for MAC.

## Background

Microcystic adnexal carcinoma (MAC) is a rare, well-differentiated cutaneous adnexal carcinoma, with local infiltration that was first described by Goldstein et al. in 1982 [1]. To date, most investigations reported MAC patients are white, with only sporadic cases in China. MAC predominantly occurs on the head or neck of middle-aged and elderly people, with 24.6% overall recurrence after surgery [2].

To decrease recurrence, surgical resection using Mohs micrographic surgery or complete circumferential periphery is the recommended standard treatment for MAC [2]. An accurate pathologic diagnosis is beneficial for the proper choice of surgical procedures and improved prognosis. Unfortunately, many patients with MAC have a crucial decline in health-related quality of life [3]. On the one hand, MAC usually presents as an asymptomatic, painless, flat nodule, without any evidence of malignant characteristics [4]; moreover, the cytopathologic features are mild and lack atypia and mitotic features. Therefore, MAC is often misdiagnosed as a benign adnexal tumor (such as syringoma and trichoepithelioma, which also usually appear on the face [5]) both clinically and pathologically [6, 7]. On the other hand, disfiguring surgical excision is used to achieve clear margins because of the deeply infiltrative growth, while infundibulocystic basal cell carcinoma, a basal cell carcinoma with histologically adnexal differentiation [8], often needs to be identified with MAC, because of its good prognosis and no need for an extended margin surgery. Both of these things lead to delayed treatment and patients suffering larger wounds.

Therefore, it is urgent to definitively explore the molecular genetic alterations in MAC to research diagnostic and therapeutic markers to achieve early diagnosis and low-trauma treatment. Two studies revealed DNA changes by next-generation sequencing. Chen et al. revealed *TP53* mutations and chromosomal loss in *cyclin-dependent kinase inhibitor 2A* (*CDKN2A*) and *cyclin-dependent kinase inhibitor 2B* (*CDKN2B*) in a metastatic MAC case [9]. Chan et al. demonstrated that inactivated *TP53* or activated JAK/STAT signaling pathways play important roles in MAC [10].

To our knowledge, there has been no transcriptome analysis of MAC. In this study, we characterized the transcriptomic alterations between 5 MAC tissues and 5 normal skin tissues at the mRNA level to identify more molecular markers for diagnosis or potential targeted therapy. The molecular markers identified from the transcriptional analysis were verified by immunohistochemistry (IHC).

## Materials and methods

### Patients and specimens

From 2017 to 2021, we searched for the patients of MAC in the pathological specimen database of sixteen grade III, class A hospitals of China, which are located in three municipalities and four provinces. Fourteen cases of MAC were found, among which five cases were consultation cases and lost contact after diagnosis, which resulted in difficulty to get the tumor tissues, two cases used up all the tumor tissues in the diagnostic process, while one case with metastasis was questioned about the pathological diagnosis. Finally, only six of them with histologically proven MAC were included in this study (defined as M1–M6). Slides of MAC were reviewed by two board-certified pathologists to confirm the diagnosis. In patients with MAC, four of the six were women, and the mean age of the patients was 51.8 years (range, 31–71 years). The follow-up information of MAC patients was obtained by telephone consultation or outpatient follow-up. The final follow-up time was November 2021, the longest follow-up time was 52 months, the shortest follow-up time was 7 months, and cases M3 and M5 failed to follow up.

Five cases of normal skin tissues were used as normal controls (defined as N1–N5). In normal control patients, four of the five were women, and the mean age was 49.6 years (range, 41–56 years). All normal control tissues were located on the cheek. We selected benign and malignant cutaneous tumors usually located in the head and face, whose histologically resemble MAC, and age of onset was similar to that of our MAC patients, for immunohistochemical verification and identification. Accordingly, five cases of syringoma (defined as S1–S5), five cases of trichoepithelioma (defined as T1–T5), and three cases of basal cell carcinoma-infundibulocystic type (defined as B1–B3) were used as of histologically similar tumor controls. In these patients, nine of the thirteen were women, and the mean age was 46.5 years (range, 16–77 years). Nine cases were located on the face, one case was on the neck, and the other three cases were located on the torso. Details of the clinical and pathological information for all patients involved in this study are shown in Supplemental Table S1. The study was approved by the ethical committee of the Sichuan Provincial People’s Hospital, and prior consent was obtained from all patients.

### RNA extractions, sequencing, and analysis

Five MAC patients (M1–M5) and five normal controls were qualified for RNA sequencing. We obtained formalin-fixed paraffin-embedded (FFPE) specimens of patients, and microdissection with laser (Leica LMD7, Germany) was used to enrich for MAC tumor cells or normal eccrine glands from approximately fifteen 8-μm-thick FFPE tissue sections of each specimen. Total RNA was extracted using the Total Nucleic Acid Isolation kit (Life Technologies, USA) according to the manufacturer’s instructions. RNA concentration was assessed by a Qubit®3.0 Fluorometer (Life Technologies, USA) and NanoDrop spectrophotometer (Thermo Fisher Scientific, USA). RNA quality was measured using an Agilent Bioanalyzer 2100 (Agilent Technologies, USA). More than 200 ng of RNA per sample was used for sequencing. RNA-seq was carried out on an Illumina NovaSeq 6000 (Illumina, USA) Next Generation Sequencing instrument at Shanghai Sinomics Corporation (China), and the average length of each raw read was 150nt.

Data was analyzed by R software (version 4.0.5) and Bioconductor packages. Differential gene expression (DGE) analysis was performed with expression levels normalized for exon length, mapped reads, and total exon fragments. In this study, we used edgeR software package to analyze the difference in gene expression between groups, by empirical Bayes method and negative binomial exact test. It executed the program of trimmed mean of M values (TMM) to eliminate the impact of different sequencing depths, and at the same time, determined the threshold of *p* value by controlling the false discovery rate (FDR) through Benjamini-Hochberg. In this process, we calculated the *p* value and performed multiple hypothesis test correction. The corrected *p* value is called the *Q* value. DGE analysis was considered differentially expressed according to the criteria (fold change ratios ≥ 2.0 or ≤ 0.5 and *Q* value < 0.05). Kyoto Encyclopedia of Genes and Genomes (KEGG) and Gene Ontology (GO) enrichment were used to infer the potential biological function of DEGs using the Database for Annotation, Visualization and Integrated Discovery (DAVID) Bioinformatics Tool (version 6.8). Results with a *p* value < 0.05 were considered significant functional categories.

### Immunohistochemistry

IHC was performed according to a two-step protocol (Dako EnVison kit, Dako, Copenhagen, Denmark) as previously described [11]. Information of the primary antibodies is listed in Supplemental Table S2. Immunohistochemical studies for CK5/6, CK20, EMA, p63, p53, p16, AR, PR, CD34, and Ki-67 were performed in M1–M6 and examined under light microscopy by two experienced pathologists. The proteins CACNA1S, MYLK3, RYR1, and ATP2A1 were verified in M1, M2, M4, M6, N1, N3, N4, and N6 (the internal control of M6), S1–S5, T1–T5, and B1–B3. M3, M5, N2, and N5 were abandoned due to insufficient tissue for further IHC analysis.

Two experienced pathologists blindly analyzed all sections under a light microscope. Based on the intensity grade of positive staining, the staining results of CACNA1S, MYLK3, RYR1, and ATP2A1 were divided into 4 categories: (–) tissue specimens without staining, (+) tissue specimens with weak staining, (++) tissue specimens with moderate staining, and (+++) tissue specimens with strong staining.

## Results

### Histopathologic characteristics and follow-up information of MACs

**Fig. 1:**
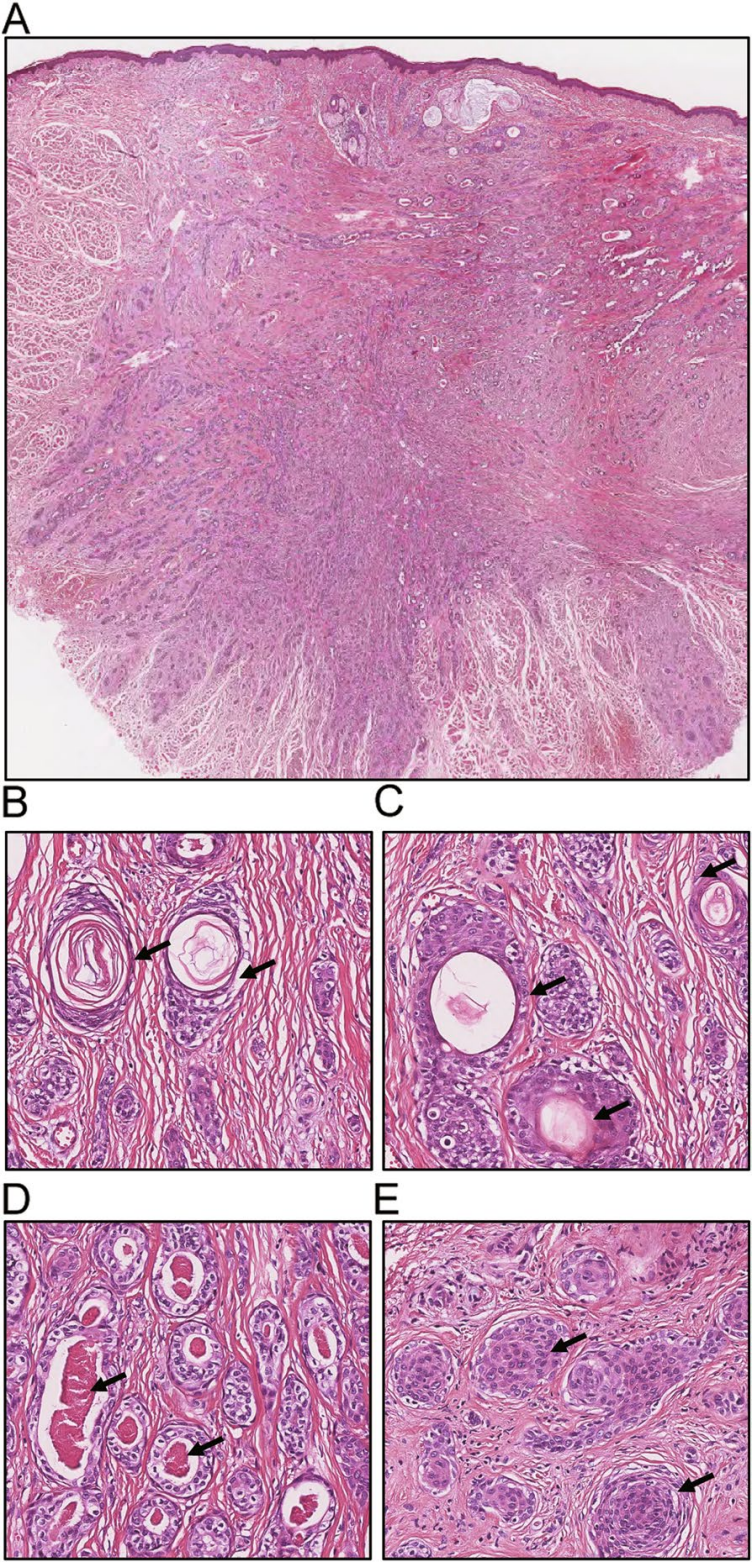
Histopathological features of MAC. MAC shows poorly circumscribed and deep infiltration into skeletal muscle (**A**, 1×). Components of keratin microcysts (**B**, black arrow), squamous differentiation, follicular differentiation (**C**, black arrow), eosinophilic secretions within the lumen (**D**, black arrow), and solid nests (**E**, black arrow) can be seen in a desmoplastic stroma. Paraffin sections were stained with HE. **B**–**D**: 200×. Abbreviations: MAC, microcystic adnexal carcinoma

All six MAC tumors showed poorly circumscribed cancer cells and deep infiltration (Fig. 1A). There were different proportions of keratin microcysts (Fig. 1B), squamous differentiation and follicular differentiation (Fig. 1C), eosinophilic secretions within the lumen (Fig. 1D), and solid nests (Fig. 1E) in the desmoplastic stroma. The cytopathologic features of all MAC tumors were very mild and lacked atypia, and mitotic features were rare. Three of them showed skeletal muscle infiltration (Supplemental Table S1). The follow-up time of M1–M6 is displayed in Table 1. There was no recurrence during the follow-up period in patients M1, M4, and M6. M2 recured in situ three times. M3 and M5 lost follow-up.

**Table 1:** Histopathologic characteristics and follow-up time of 6 cases of microcystic owed prolonged overall survivaladnexal carcinomas (MACs)

Case	Keratin microcysts	Squamous diff.	Follicular diff.	Solid component	PNI	Angiotropism	Inflammation^a^	Desmoplasia	Follow-up time (months)
M1	+	+	+	+	−	−	1	+	49
M2	+	+	−	+	−	−	2	+	52
M3	+	−	−	+	−	−	1	+	b
M4	+	+	−	+	−	−	1	+	24
M5	+	+	−	+	−	−	1	+	b
M6	−	+	−	+	−	−	1	+	7

*Abbreviations*: *diff.* differentiation, *PNI* perineural invasion, + present, − absent; ^a^inflammation was classified as 1 = minimal and 2 = mild/moderate; b: failed to follow up

### Identification of differentially expressed genes and pathway enrichment analysis

**Fig. 2:**
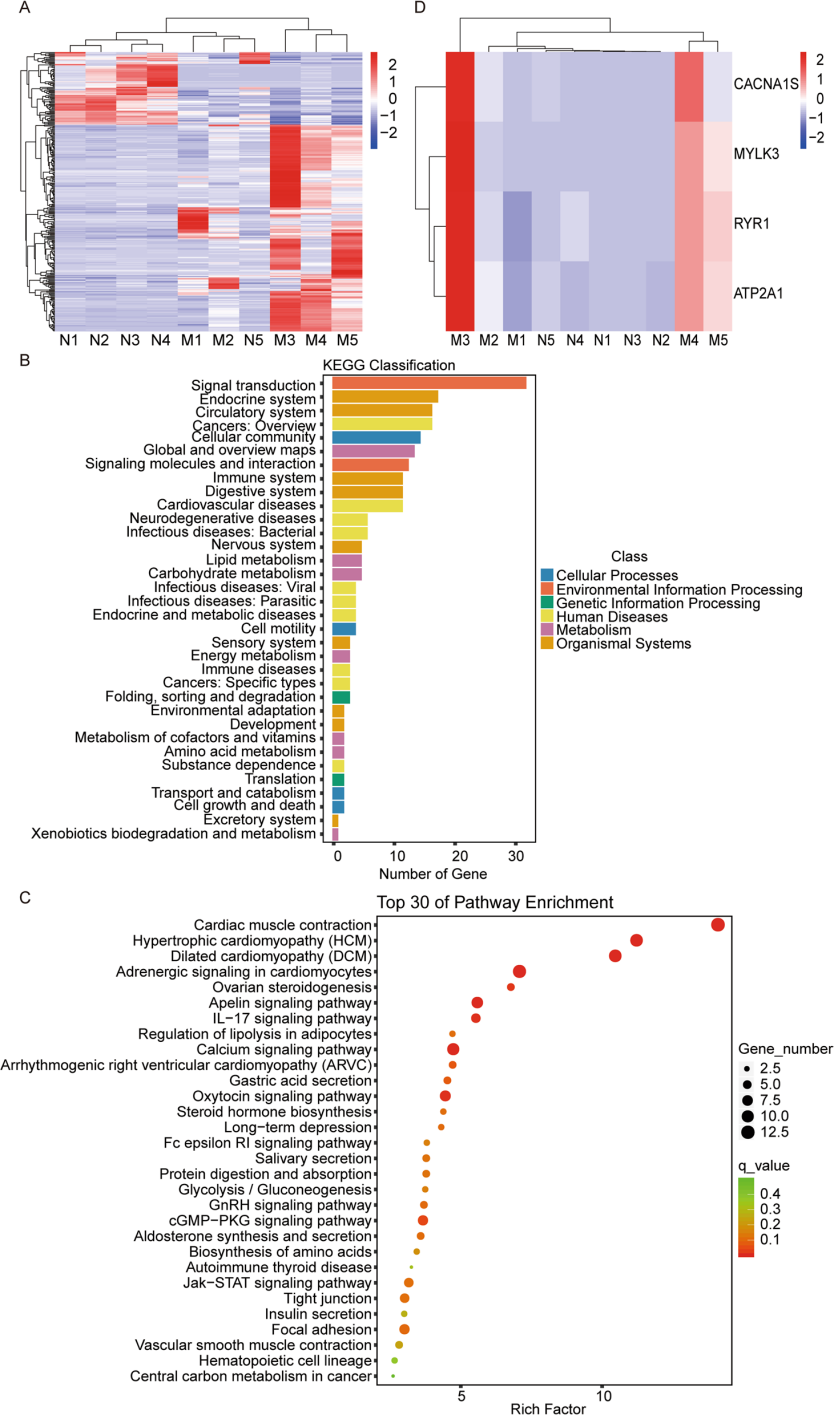
Gene expression changes in microcystic adnexal carcinoma. Five patients without metastasis and five controls qualified for RNA sequencing. **A** Heatmap shows the differentially expressed genes between MAC and normal skin tissues. **B**, **C** KEGG and GO analysis of the differentially expressed genes. **D** Heatmap of expression of the 4 key genes. Abbreviations: MAC, microcystic adnexal carcinoma tissue; N, normal skin tissue; KEGG, Kyoto Encyclopedia of Genes and Genomes; GO, Gene Ontology; *CACNA1S*, calcium voltage-gated channel subunit alpha 1S; *MYLK3*, myosin light chain kinase 3; *RYR1*, ryanodine receptor 1; *ATP2A1*, ATPase sarcoplasmic/endoplasmic reticulum Ca2+ transporting 1

We identified 304 differentially expressed genes between MAC tumor tissues and normal sweat gland control tissues by next-generation transcriptome sequencing, among which 225 genes were upregulated and 79 genes were downregulated in MAC (Fig. 2A, Supplemental Table S3). Because of the small number of cases, we did not explore the association between differentially expressed genes and patient outcomes.

Interestingly, we found that three cancer-related pathways, the JAK/STAT signaling pathway, calcium signaling pathway, and cGMP-PKG signaling pathway, were enriched by KEGG pathway enrichment analysis, indicating that they may play important roles in MAC (Fig. 2B, C). Meanwhile, we found nine genes of the calcium signaling pathway (including *ATP2A1*, *MYLK3*, *CACNA1S*, *RYR1*, *SLC25A4*, *CAMK2A*, *TNNC2*, *TNNC1*, and *MYLK2*) and seven genes of the cGMP-PKG signaling pathway (including *ATP2A1*, *MYLK3*, *CACNA1S*, *RYR1*, *SLC25A4*, *MYH7*, and *MYLK2*) were upregulated in MAC, respectively, which means the upregulated genes of the cGMP-PKG signaling pathway were included in calcium signaling-related genes except *MYH7*. Finally, we focused on four genes, *CACNA1S*, *ATP2A1*, *RYR1*, and *MYLK3*, which were commonly upregulated (over 80% patients) in MAC (Fig. 2D), for further analysis.

### Immunohistochemistry analysis

We verified several reported immunohistochemical studies in all six cases of MAC by IHC, including CK5/6, CK20, EMA, p63, p53, p16, AR, PR, CD34, and Ki-67. Most of the results were similar to those of earlier studies (Table 2, Supplemental Table S4). EMA was positive in all cases, and p53 (Supplemental Fig. S1A) showed a scattered, mottled pattern of staining (wild type) in all cases. CK5/6 (Supplemental Fig. S1B) and p63 (Supplemental Fig. S1C) were generally positive in solid nests and the basal cell layer of ductal structures. p16 was positive in five of six patients (Supplemental Fig. S1D) and was negative in case M5 (Supplemental Fig. S1E), the oldest patient (71 years old). All six MAC cancers showed a low Ki-67 index (approximately ≤ 5%) (Supplemental Fig. S1F). CK20, AR, PR, and CD34 were negative in all cases.

**Table 2:** Immunohistochemical characteristics of 6 cases of microcystic adnexal carcinomas (MACs)

Case	Protein									
	CK5/6	CK20	EMA	p63	p53	p16	AR	PR	CD34	Ki-67
M1	+	−	+	+	−^a^	+	−	−	−	3%
M2	+	−	+	+	−^a^	+	−	−	−	3%
M3	+	−	+	+	−^a^	+	−	−	−	2%
M4	+	−	+	+	−^a^	+	−	−	−	5%
M5	+	−	+	+	−^a^	−	−	−	−	5%
M6	+	−	+	+	−^a^	+	−	−	−	1%
Positive cases/total	6/6	0/6	6/6	6/6	0/6	5/6	0/6	0/6	0/6	6/6

*Abbreviations*: *Ki-67* positive index of Ki-67, + positive, − negative; ^a^scattered, mottled pattern of staining

**Fig. 3:**
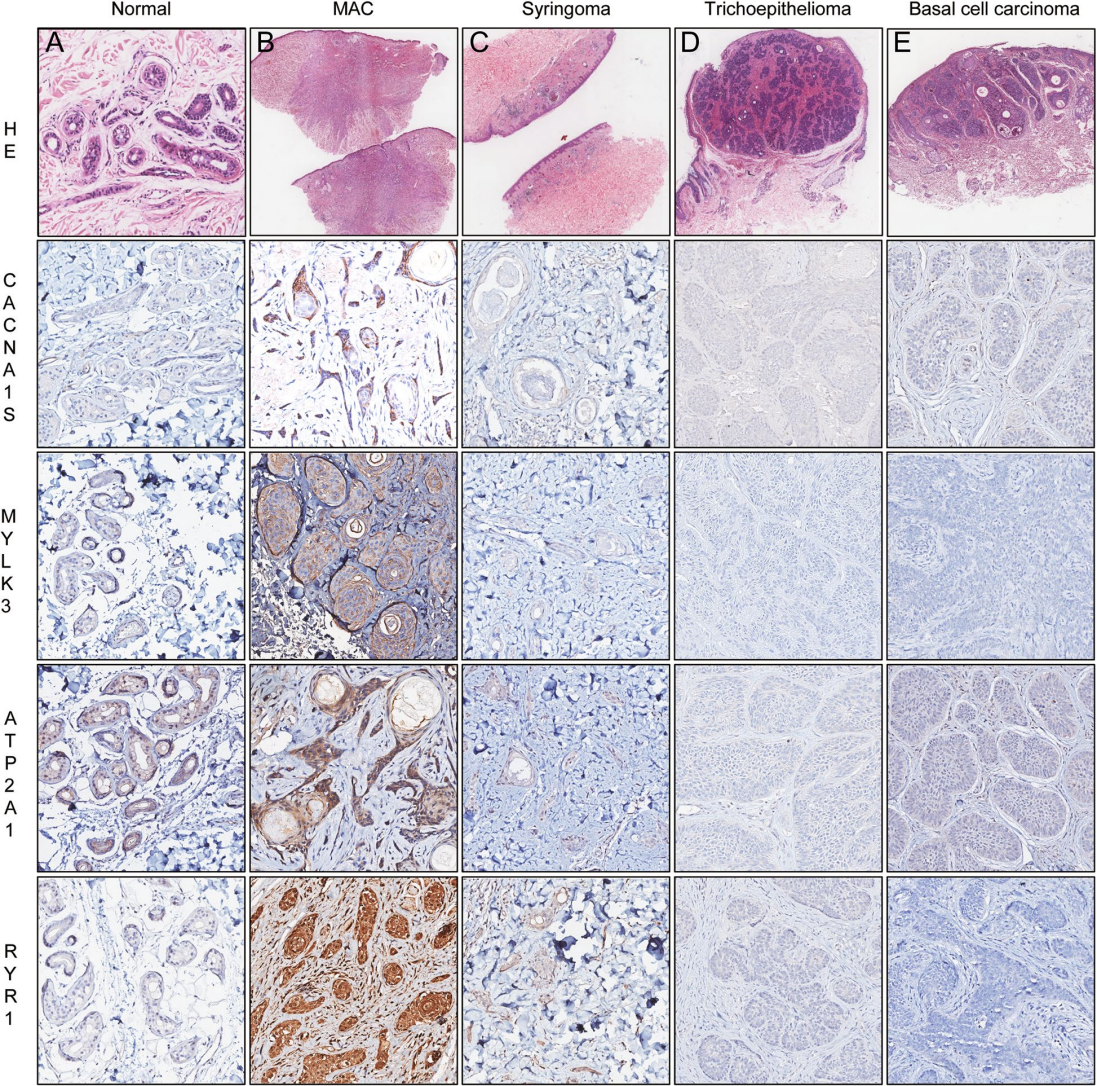
Immunohistochemical analysis of the four genes. Calcium voltage-gated channel subunit alpha 1S (CACNA1S), myosin light chain kinase 3 (MYLK3), ryanodine receptor 1 (RYR1), and ATPase sarcoplasmic/endoplasmic reticulum Ca^2+^ transporting 1 (ATP2A1) were all upregulated in MAC. HE staining pictures: **A**: 100×, **B**, **C**: 1×, **D**: 10×, **E**: 20×; IHC staining pictures: 200×

Meanwhile, we analyzed the protein expression of the four candidate genes *CACNA1S*, *ATP2A1*, *RYR1*, and *MYLK3* by IHC staining, then focused on the differences of their expression between tumor cells of MAC, syringoma, trichoepithelioma, basal cell carcinoma-infundibulocystic type and normal sweat glands. We demonstrated that the protein expression of CACNA1S, RYR1, ATP2A1, and MYLK3 was obviously upregulated in MAC tumor cells compared with normal sweat glands and syringoma tumor cells, while was basically negative in trichoepithelioma and basal cell carcinoma, infundibulocystic type (Fig. 3), except one case of trichoepithelioma showed partial RYR-1 week positive (+) (Table 3).

**Table 3:** Immunohistochemical characteristics of the four genes in MACs and their histologic mimics

Protein	MAC				Normal sweat glands				Syringoma					Trichoepithelioma					BCC		
	M1	M2	M4	M6	N1	N3	N4	N6^a^	S1	S2	S3	S4	S5	T1	T2	T3	T4	T5	B1	B2	B3
CACNA1S	+++	+++	+++	++	+	+	++	−	+	−	−	−	+	−	−	−	−	−	−	−	−
MYLK3	++	++	+	+++	+	+	−	−	++	++	+	+	−	−	−	−	−	−	−	−	−
ATP2A1	+++	+	++	++	+	+	+	+	−	−	+	−	−	−	−	−	−	−	−	−	−
RYR1	+++	+++	+++	+++	++	−	+	−	++	++	+	++	−	−	+	−	−	−	−	−	−

*Abbreviation*: *MAC* microcystic adnexal carcinoma, *BCC* basal cell carcinoma, infundibulocystic type; ^a^the internal control of M6

## Discussion

MAC is a rare skin adnexal tumor that is locally aggressive and has the potential for recurrence and metastasis. MAC is usually located on the head and neck, especially on the lip. Surgical resection using Mohs micrographic surgery or complete circumferential periphery is the standard treatment for MAC. Most MAC patients are white, with only sporadic cases in China. A systematic review reported 1968 MAC patients, the mean age was 61.8 years, and 54.1% were women [2]. In our study, all cases were located on the head and neck, and three of six were located on the lip. The mean age was 51.8 years, 66.6% (4/6) were women, and 25% (1/4) of patients suffered postoperative recurrence, based on the available follow-up information. The clinicopathologic features of MAC were first systemically discussed by Goldstein et al. in 1982 [1]. MAC usually has benign clinical characteristics and presents as asymptomatic, indurated flat plaques [12]. Microscopically, the tumor often shows both follicular [1] and sweat gland differentiation [13], occasionally dominated by one type. The cytopathologic features of MAC are mild and lack atypia and mitotic features, but it can infiltrate into the deep dermis, even skeletal muscle and peripheral nerves. Although MAC has dramatically malignant biological behavior, histologically, it may be confused with some benign skin adnexal tumors, such as desmoplastic trichoepithelioma [7, 14] and syringoma [6], especially when the biopsies are superficial, leading to delayed treatment and patients suffering larger wounds. Many studies have summarized the histological characteristics of MAC [1, 15]; however, few studies have identified the molecular genetic alterations in MAC as diagnostic and therapeutic markers to achieve early detection and treatment.

In recent decades, many studies have reported immunohistochemical studies of MAC. They revealed that carcinoembryonic antigen (CEA) [15] and CD23 [16] were positive in ductal lining cells and supported the sweat gland differentiation of MAC. CK [17, 18], CK5/6 [19], CK7 [18, 20], CK15 [21], CK19 [14], EMA [18, 22], ɑ-SMA [20], and Ln-ɤ 2 [23] were diffusely positive in most of the tumor cells; Bcl-2 [20] was focally positive; CK20 [20], c-erbB-2 [20], Ber-EP4 [14, 24], and CD34 [20] were negative, while CK20 and Ber-EP4 were positive in desmoplastic trichoepithelioma and desmoplastic basal cell carcinoma, respectively. p53 [20] was patchy and mottled, p63 [25] was positive in the periphery of tumor nests despite minimal staining within the center of the tumor islands, and adipophilin [26] was positive in the area of sebaceous differentiation, and Ki-67 usually stained less than 5%. Our study found that the expression of CK5/6, CK20, EMA, p63, p53, AR, PR, CD34, and Ki-67 in our six cases was consistent with the above results, and our results further validated the pathologic diagnosis of MAC in the six cases.

Recently, two studies proposed special molecular markers for MAC by systematically analyzing genetic changes through high-throughput sequencing at the DNA level. Chen et al. demonstrated that *TP53* mutations and chromosomal deletion of *CDKN2A* and *CDKN2B* existed in a metastatic MAC of a 68-year-old man [9]. There has been no study on the protein expression of p16 (which is encoded by *CDKN2A*) in MAC. Our study showed that p16 was scattered, mottled positive at the protein level in 5/6 cases, but was negative in M5 (follow-up information is unavailable), which was consistent with the DNA alteration results of Chen et al. in his metastatic MAC case [9]. We noticed that all 2 MAC patients who showed evidence of p16 negativity in our study and Chen et al. were older patients (68 and 71 years old). While it is known that p16 expression was upregulated along with tissue aging and therefore was considered one of the most robust aging biomarkers characterized to date [27, 28]. Several studies indicated that, as a tumor suppressor, p16 is an intrinsic human clonal evolution regulator. Its forced overexpression or downregulation impairs the progression of in vitro clonal conversion [29, 30]. In several tissues, p16 governs the processes of stem cell self-renewal and its deregulation may result in tumor development [31]. Because the data is excessively limited, the relationship between MAC metastasis, age of patients, and p16 negative expression still needs further study. Chan et al. demonstrated that inactivated *TP53* or the activated JAK/STAT signaling pathway plays important roles in MAC at the DNA level [10]. In our study, we analyzed RNA changes by next-generation transcriptome sequencing and found that the expressed genes between MAC patients and the normal population were different, and the differentially expressed genes could well distinguish the two populations. Pathway enrichment analysis found that the JAK/STAT signaling pathway (consistent with the DNA changes in Chan’s study), calcium signaling pathway, and cGMP-PKG signaling pathway have significant effects on MAC. However, we did not find mutated expression of p53 protein in all our MAC cases.

We focused our energy on calcium signaling because the JAK/STAT signaling pathway was revealed by Chan et al. The calcium signaling pathway is a cancer-associated pathway, and calcium ions (Ca^2+^) are important second messengers of varying cellular processes. Muscle contraction and hormone release as well as gene transcription are related to increasing cytosolic Ca^2+^ [32]. Ca^2+^ signaling is relevant to tumor progression, such as proliferation, migration, and apoptosis. Ca^2+^ signaling was significant in the hallmarks of cancer as described by Hanahan and Weinberg in 2000 [33] and 2011 [34]. Many proteins (channels, pumps, and exchangers) of the Ca^2+^ signaling pathway regulate cellular Ca^2+^ levels in compartments to precisely control different biological processes. The protein expression of Ca^2+^ signaling is altered in cancer, and specific cancer subtypes even manifest predominantly altered expression. It has been reported that a wide variety of proteins involved in Ca^2+^ signaling are highly expressed in malignancies, including breast, prostate, ovarian, thyroid, lung, and colon cancers [35–37]. In addition, specific Ca^2+^-permeable ion channels can cause patients to resist cancer therapies. Ca^2+^ signaling also plays a role in the tumor microenvironment. Inhibitors of Ca^2+^ signaling have undergone clinical trials and have been approved as orphan drugs for patients with solid cancers [38].

The four candidate genes (*CACNA1S*, *ATP2A1*, *RYR1*, and *MYLK3*) of Ca^2+^ signaling have special functions in tumor progression. *CACNA1S* (also named *Cav1.1*) encodes one of the five subunits of L-type voltage-gated Ca^2+^ channels, which are located in the cellular membrane and are associated with Ca^2+^ influx. Grasset et al. reported that high expression of Cav1.1 promotes the collective migration of squamous cell carcinoma cells by increasing intracellular Ca^2+^, while *Cav1.1* gene silencing by using blockers (diltiazem and verapamil) of L-type Ca^2+^ channels decreases the invasive properties of tumor cells both in vitro and in vivo [39]. *RYR1*, which is located in the endoplasmic reticulum (ER) membrane, participates in Ca^2+^ release from the ER and has a significant influence on autophagy and the activity of Ca^2+^ release-activated Ca^2+^ channels (CRACs), thus participating in the biological processes of tumors [40]. *ATP2A1*, also named *sarcoplasmic/endoplasmic reticulum Ca*^*2+*^ *ATPase 1* (*SERCA1*), is related to Ca^2+^ influx and ER refilling to assist ER Ca^2+^ levels [41] and plays a major role in muscular excitation and contraction. Chemaly et al. reported that cell apoptosis and survival were controlled by *SERCA1* through ER stress and increased Ca^2+^ levels in the cytoplasm [42], which may be a potential therapeutic target in tumors. Thapsigargin, an inhibitor of *SERCA*, has been described by Ball et al. and can effectively inhibit the function of *SERCA* [43]. MYLK3, a kinase, phosphorylates cardiac myosin heavy (*MYH7B*) and light (*MYL2*) chains, potentiating the force and rate of cross-bridge recruitment in myocytes [44]. *MYLK3* also is a member of the MAPK signaling pathway, another cancer-related signaling pathway. It was revealed that patients with *MYLK3* methylation showed prolonged overall survival in ovarian cancer treated with surgery [45]. In general, the membrane channel protein CACNA1S should locate upstream of the whole system. It works together with the ER channel proteins, ATP2A1 and RYR1; regulates the intracellular calcium concentration, then affects the concentration and function of MLYK3 downstream through the calcium/calmodulin pathway, and finally affects the formation and biological behavior of tumor cells.

To our knowledge, there have been no reports on the protein expression of CACNA1S and RYR1 in solid tumors by now. What is more, although patients with *MYLK3* methylation showed prolonged overall survival in ovarian cancer, there was no statistically significant association between expression of the gene and overall survival in these patients [45], while it is exciting that ATP2A1 mRNA and protein are overexpressed in ovarian cancer tissues compared to normal ovarian surface epithelial cell [46, 47]. Furthermore, inhibition of ATP2A1 activity by curcumin disrupts the Ca2+ homeostasis and hence promotes apoptosis in ovarian cancer cells [47]. Our transcriptomic analysis showed that *CACNA1S*, *MYLK3*, *RYR1*, and *ATP2A1* are upregulated in MAC at the mRNA level. Then, we verified protein levels of these four genes of the calcium signaling pathway by IHC because biofunction was directly occupied by proteins. Meanwhile, we also aimed to verify the value of these four candidate genes in pathologic differential diagnosis between MAC and its histological mimics. We demonstrated that the CACNA1S, MYLK3, RYR1, and ATP2A1 proteins were more highly expressed in MAC tumor cells than in normal sweat glands and syringoma, while were generally negative in tumor cells of trichoepithelioma and basal cell carcinoma, infundibulocystic type. Thus, these four candidate genes were upregulated at both the RNA and protein levels in MAC. Our findings indicated that the calcium signaling pathway may have a special influence on biological behavior in MAC, and the four genes (*CACNA1S*, *MYLK3*, *RYR1*, and *ATP2A1*) may be new diagnostic molecular markers and therapeutic targets for MAC.

The cGMP/PKG signaling pathway was also one of the enriched pathways in our study. It had revealed that activation of the cGMP/PKG pathway plays an anticancer effect in melanoma [48], head and neck squamous cell carcinoma [49], and breast [50] and colon cancer [51]. However, some studies reported opposite conclusions, and they demonstrated that activation of the cGMP/PKG signaling pathway enhances the protumorigenic effect [52, 53]. Moreover, there were complex interactions between the cGMP-PKG signaling pathway and the calcium signaling pathway. Interestingly, our results showed that the differently expressed genes were overlapped between the cGMP/PKG signaling pathway and the calcium signaling pathway in MAC, except *MYH7.* While MYH7 was also bio-functional correlated with WLYK3, one of the four genes we focused on. We put emphasis on the calcium signaling pathway other than the cGMP/PKG pathway, because the number of alternative genes was more and its *Q* value was lower than the cGMP/PKG pathway.

To our knowledge, this is the first report of transcriptional analysis of MAC worldwide. Our data entirely illustrated changes in MAC at the RNA level, but proteomic studies are still needed to confirm our results. A limitation of our study was that only five MAC cases were used for high-throughput sequencing because of the low morbidity of MAC in China. Transcriptome studies with more cases are also needed in the future.

## Conclusions

In conclusion, the four genes of the calcium signaling pathway, including *CACNA1S*, *ATP2A1*, *RYR1*, and *MYLK3*, were upregulated in MAC at the RNA level and expressed higher in MAC than in normal sweat glands and histologic mimics of MAC at the protein level. They may be new diagnostic molecular markers and therapeutic targets for MAC.

## Supplementary Information

**Additional file 1: Supplemental Fig. S1**. Immunohistochemical analysis of the reported biomarkers. p53 (A) showed a scattered mottled pattern of staining (wild type) in all cases. CK5/6 (B) and p63 (C) were generally positive in solid nests and the basal cell layer of ductal structures in primary cases. p16 was positive in five of six patients (D) and was negative in case M5 (E). All six cancers showed a low Ki-67 index (approximately < 5%, F). A-F: 200x.

**Additional file 2: Supplemental Table S1**. Clinical and pathological characteristics of patients involved in this study.

**Additional file 3: Supplemental Table S2**. Information of immunohistochemistry staining primary antibodies.

**Additional file 4: Supplemental Table S3**. Genes differentially expressed in MACs and normal sweat glands.

**Additional file 5: Supplemental Table S4**. Immunohistochemical and molecular markers that have been reported in MAC.

## Data Availability

The data that support the findings of this study are available from Sichuan Provincial People’s Hospital but restrictions apply to the availability of these data, which were used under license for the current study, and so are not publicly available. Data are however available from the authors upon reasonable request and with permission of the Sichuan Provincial People’s Hospital.

## Abbreviations

MAC: Microcystic adnexal carcinoma
DEGs: Differentially expressed genes
*CACNA1S*: *Calcium voltage-gated channel subunit alpha 1 S*
*MYLK3*: *Myosin light chain kinase 3*
*RYR1*: *Ryanodine receptor 1*
*ATP2A1*: *ATPase sarcoplasmic/endoplasmic reticulum Ca2+ transporting 1*
*CDKN2A*: *Cyclin-dependent kinase inhibitor 2A*
*CDKN2B*: *Cyclin-dependent kinase inhibitor 2B*
IHC: Immunohistochemistry
FFPE: Formalin-fixed paraffin-embedded
DGE: Differential gene expression
KEGG: Kyoto Encyclopedia of Genes and Genomes
GO: Gene Ontology
DAVID: Database for Annotation, Visualization and Integrated Discovery
CEA: Carcinoembryonic antigen
*SERCA1*: *Sarcoplasmic/endoplasmic reticulum Ca* ^*2+*^ *ATPase 1*
