# Supplementary figures and images for: Four calcium signaling pathway-related genes were upregulated in microcystic adnexal carcinoma: transcriptome analysis and immunohistochemical validation
Source: World J Surg Oncol. 2022 May 4;20:142. doi: 10.1186/s12957-022-02601-6 (PMC9066904; doi:10.1186/s12957-022-02601-6)

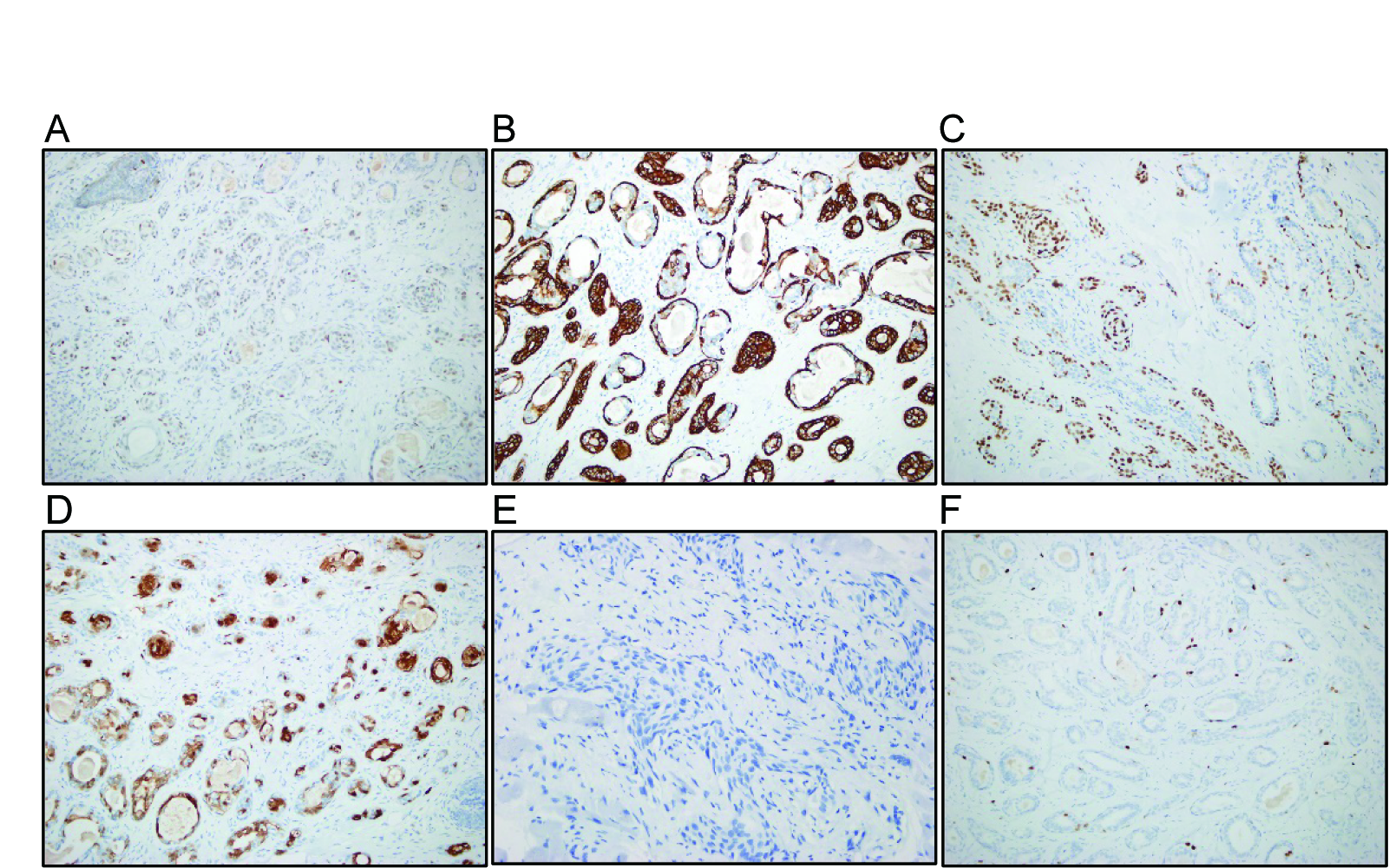

Supplement: Supplementary file 1 — Additional file 1: Supplemental Fig. S1. Immunohistochemical analysis of the reported biomarkers. p53 (A) showed a scattered mottled pattern of staining (wild type) in all cases. CK5/6 (B) and p63 (C) were generally positive in solid nests and the basal cell layer of ductal structures in primary cases. p16 was positive in five of six patients (D) and was negative in case M5 (E). All six cancers showed a low Ki-67 index (approximately < 5%, F). A-F: 200x. [file 12957_2022_2601_MOESM1_ESM.tif]
