# Supplementary material for: Four calcium signaling pathway-related genes were upregulated in microcystic adnexal carcinoma: transcriptome analysis and immunohistochemical validation
Source: World J Surg Oncol. 2022 May 4;20:142. doi: 10.1186/s12957-022-02601-6 (PMC9066904; doi:10.1186/s12957-022-02601-6)
Supplement: Supplementary file 2 — Additional file 2: Supplemental Table S1. Clinical and pathological characteristics of patients involved in this study. [file 12957_2022_2601_MOESM2_ESM.pdf]

Supplemental Table S1

Clinical and pathological characteristics of patients involved in this study.

Case	Age (years)	Gender	Ethnicity	Location	Size (mm)	Clark level	cTNM-stage
M1	66	F	C	Cheek	12.5	IV	I
M2	47	F	C	Eyebrow	6.1	V	I
M3	31	M	C	Cheek	6.0	V	I
M4	48	F	C	Upper lip	10.0	V[a]	III
M5	71	F	C	Upper lip	4.0	V[a]	III
M6	48	M	C	Lower Lip	5.2	V[a]	III
N1	66	F	C	Cheek	/	/	/
N2	47	F	C	Cheek	/	/	/
N3	31	M	C	Cheek	/	/	/
N4	48	F	C	Cheek	/	/	/
N5	71	F	C	Cheek	/	/	/
S1	25	F	C	Neck	1.5	IV	/
S2	16	M	C	Chest	2.5	IV	/
S3	46	F	C	Abdomen	1.7	IV	/
S4	27	F	C	Chest	3.2	IV	/
S5	54	F	C	Cheek	1.0	IV	/
T1	31	M	C	Nose	2.7	IV	/
T2	49	F	C	Cheek	16.6	IV	/
T3	69	F	C	Nose	7.7	IV	/
T4	72	F	C	Nose	2.6	IV	/
T5	77	M	C	Nose	4.5	IV	/
B1	34	F	C	Naso-labial groove	3.0	IV	I
B2	58	F	C	Cheek	24.0	V[a]	III
B3	47	M	C	Cheek	3.4	IV	I

Abbreviations: M: microcystic adnexal carcinomas; N: normal; S: Syringoma; T: Trichoepithelioma; B: basal cell carcinoma; F: female; M: male; C: Chinese. a: Tumor infiltrated into skeletal muscle. All patients bad no history of malignancy.
