# Supplementary material for: Four calcium signaling pathway-related genes were upregulated in microcystic adnexal carcinoma: transcriptome analysis and immunohistochemical validation
Source: World J Surg Oncol. 2022 May 4;20:142. doi: 10.1186/s12957-022-02601-6 (PMC9066904; doi:10.1186/s12957-022-02601-6)
Supplement: Supplementary file 3 — Additional file 3: Supplemental Table S2. Information of immunohistochemistry staining primary antibodies. [file 12957_2022_2601_MOESM3_ESM.pdf]

Supplemental Table S2. Information of immunohistochemistry staining primary antibodies.

Antibody	Location	Source	Clone	Dilution	ARM	Company
EMA	cytoplasm/membrane	mouse	monoclonal	ready-to-use	hot repair *	MXB
CK5/6	cytoplasm	mouse	monoclonal	ready-to-use	hot repair *	MXB
CK20	cytoplasm	mouse	monoclonal	ready-to-use	hot repair *	MXB
p16	nucleus	mouse	monoclonal	ready-to-use	hot repair *	ZSGB-BIO
p53	nucleus	mouse	monoclonal	ready-to-use	hot repair *	MXB
p63	nucleus	mouse	monoclonal	ready-to-use	hot repair *	MXB
AR	nucleus	mouse	monoclonal	ready-to-use	hot repair *	MXB
PR	nucleus	mouse	monoclonal	ready-to-use	hot repair *	Roche
CD34	membrane/cytoplasm	mouse	monoclonal	1:150	hot repair *	MXB
Ki-67	nucleus	mouse	monoclonal	ready-to-use	hot repair *	ZSGB-BIO
CACNA1S	cytoplasm	mouse	monoclonal	1:75	hot repair *	Santa Cruz
MYLK3	cytoplasm	rabbit	polyclonal	1:150	hot repair *	Proteintech
RYR1	cytoplasm	rabbit	polyclonal	1:300	hot repair *	Proteintech
ATP2A1	cytoplasm	mouse	monoclonal	1:75	hot repair *	Santa Cruz

Abbreviations: ARM: antigen retrieval method; *: hot repair, 0.1 m citric acid buffer solution, PH: 6.0, 98 °C for 10 minutes; MXB: Maixin Biotechnology, Fuzhou, China; ZSGB-BIO: Zhongshan Golden Bridge Biotechnology, Beijing, China; Roche, Basel, Switzerland; Santa Cruz, CA, USA; Proteintech, Rosemont, IL, USA.
