# Supplementary material for: Four calcium signaling pathway-related genes were upregulated in microcystic adnexal carcinoma: transcriptome analysis and immunohistochemical validation
Source: World J Surg Oncol. 2022 May 4;20:142. doi: 10.1186/s12957-022-02601-6 (PMC9066904; doi:10.1186/s12957-022-02601-6)
Supplement: Supplementary file 4 — Additional file 4: Supplemental Table S3. Genes differentially expressed in MACs and normal sweat glands. [file 12957_2022_2601_MOESM4_ESM.pdf]

Supplemental Table S3. Genes differentially expressed in MACs and normal sweat glands.

Gene Symbol	Full Title	Chromosomal Position	Log2 (Fold-change)	*P* -value	Q-value
XIRP2	xin actin binding repeat containing 2	2:166888487-167259753	NA	1.11E-11	3.95E-07
SMPX	small muscle protein X-linked	X:21705972-21758163	8.976494025	7.57E-10	1.35E-05
COL10A1	collagen type X alpha 1 chain	6:116118923-116158747	5.762589673	1.66E-09	1.59E-05
AC005520.1	novel transcript	14:73851971-73932278	6.394241625	1.79E-09	1.59E-05
MYF6	myogenic factor 6	12:80707498-80709474	NA	4.79E-09	3.42E-05
KLHL41	kelch like family member 41	2:169509702-169526262	7.524490199	6.81E-09	4.04E-05
CKM	creatine kinase, M-type	19:45306414-45322977	11.23313071	9.85E-09	5.01E-05
ANKRD1	ankyrin repeat domain 1	10:90912096-90921276	NA	1.38E-08	6.13E-05
MYL2	myosin light chain 2	12:110910819-110920722	10.34379355	2.34E-08	8.33E-05
KLHL40	kelch like family member 40	3:42685519-42692544	NA	2.18E-08	8.33E-05
MIR1-1HG	MIR1-1 host gene	20:62550453-62570764	NA	2.72E-08	8.82E-05
ACTN2	actinin alpha 2	1:236686454-236764631	6.996028127	3.19E-08	9.48E-05
PRAL	p53 regulation associated lncRNA	17:6772831-6776116	-8.10287645	3.65E-08	1.00E-04
MYPN	myopalladin	10:68106117-68212017	7.28583964	4.30E-08	0.000109384
SMYD1	SET and MYND domain containing 1	2:88067780-88113387	10.88444817	5.75E-08	0.000136556
MYOG	myogenin	1:203083132-203086036	NA	7.00E-08	0.000155861
CST1	cystatin SN	20:23747553-23751268	9.501673671	7.96E-08	0.000160677
TCAP	titin-cap	17:39664187-39666555	7.478819976	8.12E-08	0.000160677
MYH7	myosin heavy chain 7	14:23412738-23435718	12.48471051	1.14E-07	0.000184634
TTN	titin	2:178525989-178830802	6.03764692	1.07E-07	0.000184634
DCD	dermcidin	12:54644591-54648493	-5.34518826	1.14E-07	0.000184634
NEB	nebulin	2:151485336-151734487	5.91873988	1.07E-07	0.000184634
CSRP3	cysteine and glycine rich protein 3	11:19182030-19210573	10.53212738	1.22E-07	0.000189454
MYL1	myosin light chain 1	2:210290150-210315190	13.14273774	1.43E-07	0.000211655
COX6A2	cytochrome c oxidase subunit 6A2	16:31427731-31428428	11.10805571	1.56E-07	0.000213282
MB	myoglobin	22:35606764-35637951	8.409161389	1.53E-07	0.000213282
LMOD2	leiomodin 2	7:123655807-123664290	10.38925558	1.76E-07	0.000216379

MYLPF	myosin light chain, phosphorylatable, fast skeletal muscle	16:30370934-30377991	10.42220707	1.64E-07	0.000216379
DLK1	delta like non-canonical Notch ligand 1	14:100725705-100738224	NA	1.74E-07	0.000216379
MYH1	myosin heavy chain 1	17:10492307-10518543	13.03898231	1.89E-07	0.000224991
TRIM54	tripartite motif containing 54	2:27282392-27307439	8.290297994	2.00E-07	0.000229611
MMP11	matrix metallopeptidase 11	22:23768226-23784316	3.989205848	2.19E-07	0.000237906
PPP1R3A	protein phosphatase 1 regulatory subunit 3A	7:113876777-114075920	NA	2.25E-07	0.000237906
C10orf71	chromosome 10 open reading frame 71	10:49299193-49327487	10.13876311	2.27E-07	0.000237906
AQP5	aquaporin 5	12:49961870-49965681	-3.47645152	2.49E-07	0.000247656
NRAP	nebulin related anchoring protein	10:113588716-113664127	9.371472798	2.50E-07	0.000247656
CACNG1	calcium voltage-gated channel auxiliary subunit gamma 1	17:67044590-67056797	NA	3.97E-07	0.000374718
ASB15	ankyrin repeat and SOCS box containing 15	7:123567010-123639481	NA	4.06E-07	0.000374718
ASB5	ankyrin repeat and SOCS box containing 5	4:176213673-176277571	9.953232528	4.10E-07	0.000374718
TNNT3	troponin T3, fast skeletal type	11:1919562-1938706	6.456566358	5.19E-07	0.000462378
CYP19A1	cytochrome P450 family 19 subfamily A member 1	15:51208057-51338610	5.861727766	5.47E-07	0.00046422
MYH2	myosin heavy chain 2	17:10521148-10549957	10.94180782	5.66E-07	0.00046935
MYHAS	myosin heavy chain gene cluster antisense RNA	17:10383144-10623886	NA	6.26E-07	0.000506681
LGALSL	galectin like	2:64453969-64461381	-3.05522937	6.92E-07	0.000548013
OBSCN	obscurin, cytoskeletal calmodulin and titin-interacting RhoGEF	1:228208130-228378874	2.758573826	8.53E-07	0.000646399
STAC3	SH3 and cysteine rich domain 3	12:57243453-57251193	4.920492789	8.49E-07	0.000646399
ACTA1	actin, alpha 1, skeletal muscle	1:229431245-229434098	8.120592007	8.91E-07	0.000661595
PSG3	pregnancy specific beta-1-glycoprotein 3	19:42721638-42740569	NA	9.33E-07	0.000678074
CGB5	chorionic gonadotropin subunit beta 5	19:49043884-49045311	8.983015241	1.08E-06	0.000767895
XIRP1	xin actin binding repeat containing 1	3:39183210-39192596	7.430039999	1.36E-06	0.000952649
NMRK2	nicotinamide riboside kinase 2	19:3933103-3942416	NA	1.50E-06	0.001007988
Z97200.1	novel transcript	1:170667381-170669425	4.227119711	1.50E-06	0.001007988
CACNG6	calcium voltage-gated channel auxiliary subunit gamma 6	19:53992288-54012669	9.647494231	1.54E-06	0.001016436
JPT2	Jupiter microtubule associated homolog 2	16:1678256-1702280	-3.28492306	1.64E-06	0.00106449
CSF3R	colony stimulating factor 3 receptor	1:36466043-36483278	3.232384458	1.74E-06	0.001102489
MYOZ1	myozenin 1	10:73631654-73641757	7.551929212	1.76E-06	0.001102489
MYBPC2	myosin binding protein C, fast type	19:50432903-50466321	8.580519184	2.00E-06	0.001206049
ATP2A1	ATPase sarcoplasmic/endoplasmic reticulum Ca2+ transporting 1	16:28878405-28904509	8.04785328	1.97E-06	0.001206049
EEF1A2	eukaryotic translation elongation factor 1 alpha 2	20:63488013-63499315	6.34394535	2.07E-06	0.001229253

CGA	glycoprotein hormones, alpha polypeptide	6:87085498-87095406	9.278633962	2.47E-06	0.001441348
ADAM12	ADAM metallopeptidase domain 12	10:126012381-126388455	3.62559338	2.52E-06	0.00144801
MYLK3	myosin light chain kinase 3	16:46703369-46790407	7.046523485	2.59E-06	0.001462551
TFPI2	tissue factor pathway inhibitor 2	7:93885397-93890991	7.432951052	2.92E-06	0.001622803
ISM2	isthmin 2	14:77474394-77498850	7.39539989	3.36E-06	0.001834416
TNNC2	troponin C2, fast skeletal type	20:45823214-45833745	7.870772769	3.43E-06	0.001834416
SPP1	secreted phosphoprotein 1	4:87975650-87983426	3.954515578	3.45E-06	0.001834416
CAMK2A	calcium/calmodulin dependent protein kinase II alpha	5:150219491-150290291	4.123640369	3.61E-06	0.001888769
TNNI2	troponin I2, fast skeletal type	11:1838989-1841680	5.762108083	4.12E-06	0.002037479
METTL21EP	methyltransferase like 21E, pseudogene	13:102880099-102896033	6.141037075	4.05E-06	0.002037479
MIR133A1HG	MIR133A1 host gene	18:21825487-21831410	7.045373601	4.07E-06	0.002037479
AC068506.1	novel transcript	18:34892013-34902885	NA	4.02E-06	0.002037479
PYGM	glycogen phosphorylase, muscle associated	11:64746389-64760297	6.028321972	4.43E-06	0.002161887
MYBPC1	myosin binding protein C, slow type	12:101568353-101686018	6.67920158	4.59E-06	0.002208165
AP000295.1	novel protein	21:33246774-33283212	NA	4.72E-06	0.002244324
RPTN	repetin	1:152153595-152159228	-6.43914425	5.32E-06	0.002495219
SHISA2	shisa family member 2	13:26044597-26051031	3.133048204	5.50E-06	0.002545147
DEFB103A	defensin beta 103A	8:7881204-7882664	-6.76044191	5.86E-06	0.002676958
NKX6-1	NK6 homeobox 1	4:84491987-84498450	7.710964277	5.99E-06	0.002699123
CAV3	caveolin 3	3:8733800-8841808	7.168696292	6.21E-06	0.002763666
CBLN4	cerebellin 4 precursor	20:55997440-56005472	6.837756721	6.65E-06	0.002908557
CPM	carboxypeptidase M	12:68842197-68971570	-2.77543184	6.78E-06	0.002908557
CGB8	chorionic gonadotropin subunit beta 8	19:49047638-49049106	10.26872631	6.70E-06	0.002908557
DUSP26	dual specificity phosphatase 26	8:33591332-33600106	7.705170154	7.47E-06	0.003131359
PGAM2	phosphoglycerate mutase 2	7:44062727-44065587	6.932536682	7.44E-06	0.003131359
SLN	sarcolipin	11:107707378-107719693	5.787934307	7.66E-06	0.003171407
MIER1	MIER1 transcriptional regulator	1:66924895-66988619	3.115225343	7.82E-06	0.00320035
CHGB	chromogranin B	20:5911430-5925361	11.36619703	8.31E-06	0.003281513
TNNC1	troponin C1, slow skeletal and cardiac type	3:52451102-52454070	6.248564888	8.23E-06	0.003281513
S100A7A	S100 calcium binding protein A7A	1:153416524-153423225	-5.05480508	8.38E-06	0.003281513
AL137246.2	novel transcript	13:102903339-102904000	7.365616898	8.14E-06	0.003281513
COMP	cartilage oligomeric matrix protein	19:18782773-18791314	3.650122689	8.91E-06	0.003417153

PCDHGA1	protocadherin gamma subfamily A, 1	5:141330571-141512981	-5.5365562	8.92E-06	0.003417153
CLDN6	claudin 6	16:3014712-3020071	NA	1.01E-05	0.003775135
TPM2	tropomyosin 2	9:35681992-35691020	4.341504084	9.99E-06	0.003775135
CSH2	chorionic somatomammotropin hormone 2	17:63872012-63873766	11.2231196	1.02E-05	0.003778823
APOBEC2	apolipoprotein B mRNA editing enzyme catalytic subunit 2	6:41053304-41064511	7.259889102	1.08E-05	0.003951629
KRT26	keratin 26	17:40766238-40772162	NA	1.12E-05	0.004065837
CMYA5	cardiomyopathy associated 5	5:79689877-79800240	3.872156405	1.14E-05	0.004119109
CASQ1	calsequestrin 1	1:160190556-160201886	6.908679839	1.17E-05	0.00415913
KRTAP2-4	keratin associated protein 2-4	17:41065116-41065879	-9.27035168	1.22E-05	0.004266916
AC018521.1	novel transcript	17:47945424-47981736	NA	1.22E-05	0.004266916
CHRNA1	cholinergic receptor nicotinic alpha 1 subunit	2:174747592-174787935	4.768176682	1.29E-05	0.004425032
CRH	corticotropin releasing hormone	8:66176382-66178725	NA	1.30E-05	0.004425032
CCL18	C-C motif chemokine ligand 18	17:36064280-36072032	-3.52821626	1.30E-05	0.004425032
PAX7	paired box 7	1:18631006-18748866	NA	1.32E-05	0.004425355
KRTAP2-2	keratin associated protein 2-2	17:41054498-41055230	NA	1.50E-05	0.00498644
DHRS7C	dehydrogenase/reductase 7C	17:9771434-9791297	NA	1.54E-05	0.005076855
TRDN	triadin	6:123216339-123637093	7.553554203	1.57E-05	0.005123511
GH2	growth hormone 2	17:63880215-63881935	NA	1.61E-05	0.005207649
COX6B2	cytochrome c oxidase subunit 6B2	19:55349306-55354814	3.977872619	1.64E-05	0.005255644
IL4	interleukin 4	5:132673986-132682676	NA	1.66E-05	0.005285779
LAMP5	lysosomal associated membrane protein family member 5	20:9514358-9530524	3.059071391	1.75E-05	0.005503777
S100A8	S100 calcium binding protein A8	1:153390032-153391188	-3.54801007	1.77E-05	0.005519756
SLC36A2	solute carrier family 36 member 2	5:151314978-151347590	6.496706668	1.84E-05	0.00559739
AL136295.1	novel protein	14:24189157-24213473	NA	1.83E-05	0.00559739
AC015878.1	uncharacterized LOC101927521	18:21380286-21451017	NA	1.84E-05	0.00559739
GJB2	gap junction protein beta 2	13:20187470-20192898	-2.9390756	1.92E-05	0.005758217
KRT28	keratin 28	17:40792203-40799959	-5.24237501	1.92E-05	0.005758217
AC020909.2	novel transcript	19:50486810-50487638	7.403436361	1.99E-05	0.005914075
LCMT1-AS2	LCMT1 antisense RNA 2	16:25140577-25149032	8.030039045	2.03E-05	0.005967619
LINCMD1	long intergenic non-protein coding RNA, muscle differentiation 1	6:52146814-52151119	NA	2.12E-05	0.006196424
MYH4	myosin heavy chain 4	17:10443290-10469559	NA	2.25E-05	0.006507919
AC008763.3	novel protein	19:7678501-7682854	NA	2.28E-05	0.006544851

CPNE9	copine family member 9	3:9703807-9729908	4.341357649	2.33E-05	0.006644958
COL8A1	collagen type VIII alpha 1 chain	3:99638475-99799226	2.851989494	2.47E-05	0.006995253
MYOT	myotilin	5:137867791-137887851	6.975021868	2.65E-05	0.007400309
ERVW-1	endogenous retrovirus group W member 1, envelope	7:92468380-92477986	6.98675504	2.67E-05	0.007400309
C4orf54	chromosome 4 open reading frame 54	4:99636529-99654648	8.797817191	2.68E-05	0.007400309
MUC5B	mucin 5B, oligomeric mucus/gel-forming	11:1223066-1262172	4.565497123	2.71E-05	0.007432605
PCDHGA4	protocadherin gamma subfamily A, 4	5:141355025-141512979	8.169143264	2.89E-05	0.007849143
RGS4	regulator of G protein signaling 4	1:163068775-163076802	4.263520948	2.91E-05	0.007860954
KRTAP4-4	keratin associated protein 4-4	17:41159651-41160731	NA	2.97E-05	0.007938287
SEMA4A	semaphorin 4A	1:156147366-156177752	-2.80910662	2.99E-05	0.007938287
PVALB	parvalbumin	22:36800684-36819479	9.858810612	3.01E-05	0.007941631
FAP	fibroblast activation protein alpha	2:162170684-162245151	2.341845596	3.14E-05	0.008218762
CACNA1S	calcium voltage-gated channel subunit alpha1 S	1:201039512-201112566	5.677863216	3.23E-05	0.008387416
PADI3	peptidyl arginine deiminase 3	1:17249098-17284233	3.965447062	3.30E-05	0.008523778
TRIM72	tripartite motif containing 72	16:31214021-31231537	4.305430066	3.37E-05	0.008637169
CYP11A1	cytochrome P450 family 11 subfamily A member 1	15:74337759-74367740	5.269705424	3.46E-05	0.008815885
TPM1	tropomyosin 1	15:63042632-63071915	3.372227973	3.69E-05	0.009255278
LMOD3	leiomodin 3	3:69106872-69123032	7.17788268	3.78E-05	0.00942273
ANK1	ankyrin 1	8:41653220-41896762	4.417153079	3.91E-05	0.009542425
STMN2	stathmin 2	8:79610814-79666175	-2.28236873	3.88E-05	0.009542425
AS3MT	arsenite methyltransferase	10:102869516-102901899	NA	3.89E-05	0.009542425
SPTSSB	serine palmitoyltransferase small subunit B	3:161344792-161372880	-2.19302398	4.06E-05	0.009851117
ADIPOQ	adiponectin, C1Q and collagen domain containing	3:186842690-186858463	-4.90035935	4.10E-05	0.009864709
PLAC4	placenta specific 4	21:41175231-41180818	7.07007907	4.17E-05	0.009978714
FAM57B	family with sequence similarity 57 member B	16:30024427-30052978	5.57311299	4.26E-05	0.010123794
MYOM1	myomesin 1	18:3066807-3220108	3.667667589	4.36E-05	0.010254161
FNDC1	fibronectin type III domain containing 1	6:159169397-159272109	2.73911464	4.37E-05	0.010254161
PAGE4	PAGE family member 4	X:49829260-49833973	NA	4.49E-05	0.010441986
FBXO40	F-box protein 40	3:121593119-121630295	6.506753567	4.57E-05	0.010441986
KRTAP4-8	keratin associated protein 4-8	17:41096981-41098141	-8.50409426	4.52E-05	0.010441986
LOXL4	lysyl oxidase like 4	10:98247690-98268250	3.086719009	4.75E-05	0.010785466
AL499627.1	novel transcript	20:62513909-62516096	7.000521352	4.81E-05	0.01085593

AL138759.1	novel transcript	10:94577439-94611238	NA	5.20E-05	0.011642792
TENT2	terminal nucleotidyltransferase 2	5:79612120-79686648	-2.55926176	5.26E-05	0.011722504
TNNI1	troponin I1, slow skeletal type	1:201403768-201429866	4.843726886	5.35E-05	0.011787974
SMTNL2	smoothelin like 2	17:4583999-4608319	4.983210051	5.36E-05	0.011787974
NAA40	N(alpha)-acetyltransferase 40, NatD catalytic subunit	11:63938959-63957328	3.202145189	5.52E-05	0.012014581
PRR9	proline rich 9	1:153217584-153219317	-4.06060855	5.53E-05	0.012014581
RBM24	RNA binding motif protein 24	6:17281346-17293875	3.646298459	5.80E-05	0.012264098
KSR1	kinase suppressor of ras 1	17:27456714-27626438	3.280663502	5.71E-05	0.012264098
ACTC1	actin, alpha, cardiac muscle 1	15:34788096-34796139	4.676477227	5.78E-05	0.012264098
DEFB4A	defensin beta 4A	8:7894629-7896711	-7.40228423	5.79E-05	0.012264098
C10orf99	chromosome 10 open reading frame 99	10:84173738-84185294	-3.29800706	5.82E-05	0.012264098
ENO3	enolase 3	17:4948092-4957131	5.407844095	6.07E-05	0.012663265
PSG9	pregnancy specific beta-1-glycoprotein 9	19:43211791-43269530	NA	6.08E-05	0.012663265
PSG8	pregnancy specific beta-1-glycoprotein 8	19:42752686-42855691	NA	6.15E-05	0.012665387
BTN3A2	butyrophilin subfamily 3 member A2	6:26365159-26378320	3.103028988	6.13E-05	0.012665387
UNC45B	unc-45 myosin chaperone B	17:35147817-35189345	6.445400638	6.40E-05	0.013108249
ACPP	acid phosphatase, prostate	3:132317367-132368298	-2.25356635	6.63E-05	0.013298015
SRL	sarcalumenin	16:4189374-4242080	5.358809245	6.60E-05	0.013298015
KRTAP3-1	keratin associated protein 3-1	17:41008521-41019324	-6.81617231	6.61E-05	0.013298015
MRLN	myoregulin	10:59736692-59756041	6.961287364	6.64E-05	0.013298015
RPL3L	ribosomal protein L3 like	16:1943974-1957606	5.826654471	6.78E-05	0.013495455
CSF2	colony stimulating factor 2	5:132073790-132076170	NA	6.85E-05	0.013557567
KRTAP16-1	keratin associated protein 16-1	17:41307700-41309253	NA	6.90E-05	0.013578842
JAG2	jagged 2	14:105140981-105168824	-2.10497799	7.08E-05	0.013853148
PSG1	pregnancy specific beta-1-glycoprotein 1	19:42866464-42879822	7.257918967	7.13E-05	0.013873238
MYH13	myosin heavy chain 13	17:10300865-10373130	NA	7.33E-05	0.014199803
KRT35	keratin 35	17:41476689-41481140	-4.98708481	7.37E-05	0.014200633
MYLK2	myosin light chain kinase 2	20:31819308-31834689	6.239579694	7.48E-05	0.014250706
PCDHGB5	protocadherin gamma subfamily B, 5	5:141397987-141512979	-6.14125482	7.45E-05	0.014250706
ANKRD13A	ankyrin repeat domain 13A	12:109999186-110039763	-2.30989351	7.59E-05	0.01432824
TGM2	transglutaminase 2	20:38127387-38166578	5.22307181	7.60E-05	0.01432824
CSH1	chorionic somatomammotropin hormone 1	17:63894909-63896661	9.30072243	7.84E-05	0.014694899

LINC02544	long intergenic non-protein coding RNA 2544	6:169175304-169182740	6.268241969	8.13E-05	0.015087172
ERVH48-1	endogenous retrovirus group 48 member 1	21:42916803-42925646	6.524704101	8.30E-05	0.015314951
GLCE	glucuronic acid epimerase	15:69160584-69272217	-2.79791157	8.70E-05	0.015899136
HJV	hemojuvelin BMP co-receptor	1:146017468-146036746	7.093796104	8.99E-05	0.016257674
KRTAP9-4	keratin associated protein 9-4	17:41249687-41250653	-6.72225866	8.95E-05	0.016257674
ATP1A2	ATPase Na+/K+ transporting subunit alpha 2	1:160115759-160143591	3.958402173	9.05E-05	0.016280166
MYL3	myosin light chain 3	3:46857872-46882169	4.846534991	9.31E-05	0.016661355
SERPINE1	serpin family E member 1	7:101127089-101139266	3.215214925	9.62E-05	0.017128512
KRTAP2-1	keratin associated protein 2-1	17:41046541-41047316	-6.99908644	9.79E-05	0.017347618
COL21A1	collagen type XXI alpha 1 chain	6:56056590-56394094	2.9088391	0.00010058	0.017739789
ERVV-1	endogenous retrovirus group V member 1, envelope	19:53013921-53016122	NA	0.00010208	0.017914846
AZGP1	alpha-2-glycoprotein 1, zinc-binding	7:99966720-99976157	-1.86765712	0.00010392	0.018143289
ISL1	ISL LIM homeobox 1	5:51383391-51394738	6.581826514	0.0001114	0.019266591
MYOZ3	myozenin 3	5:150660874-150679365	3.828771752	0.00011224	0.019318232
TCHH	trichohyalin	1:152106317-152115454	-4.19741403	0.00011654	0.019961469
SLCO1A2	solute carrier organic anion transporter family member 1A2	12:21264600-21419594	7.141149974	0.00011995	0.020427375
KRTAP3-2	keratin associated protein 3-2	17:40999193-40999894	-7.07353413	0.00012041	0.020427375
TNNT1	troponin T1, slow skeletal type	19:55132794-55149354	5.047863203	0.00012202	0.020602927
NOX4	NADPH oxidase 4	11:89324356-89498187	3.378366494	0.00012502	0.020813748
JSRP1	junctional sarcoplasmic reticulum protein 1	19:2252252-2269759	5.531437228	0.0001242	0.020813748
ADAM1A	ADAM metallopeptidase domain 1A (pseudogene)	12:111899263-111901391	5.25901225	0.00012464	0.020813748
WT1	Wilms tumor 1	11:32387775-32435630	8.82121901	0.00012829	0.021258056
SPRR2A	small proline rich protein 2A	1:153056113-153057537	-4.70315331	0.0001346	0.022098217
ABRA	actin binding Rho activating protein	8:106759483-106770245	5.927106155	0.0001371	0.022404925
DEFB103B	defensin beta 103B	8:7428888-7430348	-6.41280434	0.00013801	0.02245074
KCNK5	potassium two pore domain channel subfamily K member 5	6:39188973-39229450	-2.11737646	0.00014016	0.022698105
VGLL1	vestigial like family member 1	X:136532152-136556807	5.979186511	0.00014675	0.023580711
CGB2	chorionic gonadotropin subunit beta 2	19:49031912-49033238	4.800005325	0.00014694	0.023580711
MDK	midkine	11:46380756-46383837	2.2597395	0.00015196	0.024278191
PTGER3	prostaglandin E receptor 3	1:70852353-71047808	-2.09209582	0.00015467	0.024600004
GLTP	glycolipid transfer protein	12:109850943-109880488	-2.06358055	0.00015556	0.0246076
MAL2	mal, T cell differentiation protein 2 (gene/pseudogene)	8:119165034-119245673	-1.96323285	0.00015651	0.0246076

PLIN1	perilipin 1	15:89664365-89679427	-3.70828624	0.00015679	0.0246076
KRTAP10-3	keratin associated protein 10-3	21:44557790-44558760	-7.09540159	0.00015876	0.024699766
KISS1	KiSS-1 metastasis suppressor	1:204190341-204196486	9.259368001	0.00016235	0.025148246
CAVIN4	caveolae associated protein 4	9:100578079-100587906	6.318483632	0.0001668	0.025614702
KRTAP4-9	keratin associated protein 4-9	17:41105332-41106488	-6.32397082	0.00016642	0.025614702
XAGE3	X antigen family member 3	X:52862525-52868068	NA	0.00017331	0.026500459
KRTAP9-3	keratin associated protein 9-3	17:41232463-41233454	-6.18045256	0.00017536	0.026698471
SH3BGR	SH3 domain binding glutamate rich protein	21:39445855-39515506	4.807161668	0.00017805	0.026992728
CGB3	chorionic gonadotropin subunit beta 3	19:49022869-49024333	9.413177514	0.0001806	0.027242019
INHBA	inhibin subunit beta A	7:41667168-41705834	2.830944074	0.00018122	0.027242019
FSD2	fibronectin type III and SPRY domain containing 2	15:82755362-82806070	5.908776179	0.00018825	0.028179151
MTND1P23	MT-ND1 pseudogene 23	1:629062-629433	5.360917617	0.00019289	0.028752932
LRTM1	leucine rich repeats and transmembrane domains 1	3:54918237-54967088	10.16313659	0.000197	0.029244251
H19	H19, imprinted maternally expressed transcript	11:1995163-2001470	4.410897326	0.00019923	0.029330245
KRTAP5-4	keratin associated protein 5-4	11:1620958-1622138	NA	0.00019869	0.029330245
KRTAP1-3	keratin associated protein 1-3	17:41033884-41034855	-6.29749828	0.00020313	0.029781783
HOXB8	homeobox B8	17:48611377-48614939	6.581845895	0.00020633	0.030003143
GDF15	growth differentiation factor 15	19:18374731-18389176	5.850788913	0.00020564	0.030003143
C9orf135	chromosome 9 open reading frame 135	9:69820793-69906232	NA	0.00020868	0.030222371
KRT85	keratin 85	12:52360006-52367481	-4.2104625	0.00021845	0.031509494
GPR12	G protein-coupled receptor 12	13:26755200-26760785	-5.18443698	0.00022262	0.031725582
PDLIM3	PDZ and LIM domain 3	4:185500660-185535612	4.398092798	0.0002219	0.031725582
MYOD1	myogenic differentiation 1	11:17719568-17722131	7.833271258	0.00022424	0.031793557
CSHL1	chorionic somatomammotropin hormone like 1	17:63909597-63918838	NA	0.00022578	0.031793557
SLC25A4	solute carrier family 25 member 4	4:185143241-185150382	3.386570342	0.00022843	0.032039717
PFKM	phosphofructokinase, muscle	12:48105139-48146404	2.683585991	0.00023138	0.032326561
KRTAP4-12	keratin associated protein 4-12	17:41123091-41124167	-6.67367244	0.00023621	0.032873418
KRTAP19-5	keratin associated protein 19-5	21:30501657-30502117	NA	0.00024753	0.03431074
MTATP8P1	MT-ATP8 pseudogene 1	1:633535-633741	-7.00954454	0.00024847	0.03431074
U82695.1	novel pseudogene	X:153479266-153487088	6.594933931	0.00025005	0.034395795
S100A12	S100 calcium binding protein A12	1:153373706-153375649	-7.39909006	0.00025318	0.034692696
AGPAT3	1-acylglycerol-3-phosphate O-acyltransferase 3	21:43865186-43986536	-2.33074717	0.00025932	0.035397251

JPH1	junctophilin 1	8:74234700-74321328	4.054368797	0.00026437	0.035668473
IL31RA	interleukin 31 receptor A	5:55851379-55922853	3.519593053	0.00026519	0.035668473
HTRA4	HtrA serine peptidase 4	8:38974164-38988662	5.875462958	0.00026576	0.035668473
AC020637.1	uncharacterized LOC100506869	12:58544124-58813060	NA	0.00026631	0.035668473
AP005212.4	novel transcript	18:14225224-14342505	5.081326403	0.00026481	0.035668473
NEURL1	neuralized E3 ubiquitin protein ligase 1	10:103493979-103592552	2.869071063	0.00027048	0.036036185
SLC22A11	solute carrier family 22 member 11	11:64555626-64572875	6.527094131	0.00027863	0.036824808
CTRB2	chymotrypsinogen B2	16:75204096-75207185	NA	0.00027908	0.036824808
DDIT4L	DNA damage inducible transcript 4 like	4:100185870-100190782	3.513896432	0.0002804	0.036862119
CDH1	cadherin 1	16:68737225-68835548	-2.86656369	0.00028597	0.037456308
PSG2	pregnancy specific beta-1-glycoprotein 2	19:43064211-43083045	7.894057578	0.0002899	0.037833062
AL157392.5	novel protein	10:13610047-13655929	9.226245865	0.00029142	0.037892344
HAPLN1	hyaluronan and proteoglycan link protein 1	5:83637805-83721613	3.593021154	0.00030366	0.039197195
SFRP4	secreted frizzled related protein 4	7:37905932-38025695	4.441166136	0.00030629	0.039357666
NCCRP1	non-specific cytotoxic cell receptor protein 1 homolog (zebrafish)	19:39196961-39201884	-2.17502152	0.00030711	0.039357666
KLHL31	kelch like family member 31	6:53647901-53665708	3.039374782	0.0003129	0.039955387
LINC01405	long intergenic non-protein coding RNA 1405	12:110936585-110958208	5.857126297	0.00031808	0.040472444
DUSP13	dual specificity phosphatase 13	10:75094432-75109221	5.067868376	0.00032992	0.04182879
TXLNB	taxilin beta	6:139240061-139292139	3.191406939	0.00033484	0.042153531
KRTAP4-1	keratin associated protein 4-1	17:41184102-41185342	-7.06201165	0.0003338	0.042153531
CNFN	cornifelin	19:42387019-42390287	-2.79351366	0.00033789	0.04238776
NOTUM	notum, palmitoleoyl-protein carboxylesterase	17:81952507-81961840	4.061972606	0.00033941	0.042428142
PLA2G4D	phospholipase A2 group IVD	15:42067009-42094554	-2.95949788	0.0003433	0.042764811
GNLY	granulysin	2:85685175-85698854	5.053991797	0.00034839	0.043224899
RYR1	ryanodine receptor 1	19:38433699-38587564	4.181793808	0.0003505	0.043224899
AC245297.1	phosphodiesterase 4D interacting protein-like	1:149197992-149321732	4.969673813	0.00035063	0.043224899
KRTAP9-9	keratin associated protein 9-9	17:41255384-41256364	NA	0.00035205	0.043250382
CA12	carbonic anhydrase 12	15:63321378-63382161	-1.52278581	0.00035446	0.043396254
MMP13	matrix metallopeptidase 13	11:102942995-102955734	6.110360314	0.00035905	0.043702319
AL358781.1	uncharacterized LOC107987135	9:131497479-131500191	9.554142421	0.00035941	0.043702319
KRTAP4-3	keratin associated protein 4-3	17:41167231-41168194	-6.27630636	0.00036376	0.044080478
KRTAP9-8	keratin associated protein 9-8	17:41238045-41239004	NA	0.00036595	0.044179741

AL353997.3	CMT1A duplicated region transcript 15-like 2 (CDRT15L2) pseudogene	17:18388871-18389459	-4.06855604	0.00036706	0.044179741
UGT1A8	UDP glucuronosyltransferase family 1 member A8	2:233617645-233773310	NA	0.00037135	0.044294036
AL121928.1	novel transcript, antisense to ACTR1A	10:102483039-102483559	NA	0.00037174	0.044294036
ANO5	anoctamin 5	11:22193176-22283357	3.996073113	0.00037309	0.044307359
PAPPA2	pappalysin 2	1:176463171-176845605	4.672773253	0.00037482	0.044364859
CLCN1	chloride voltage-gated channel 1	7:143316126-143352083	5.792820598	0.0003799	0.04481723
PSG6	pregnancy specific beta-1-glycoprotein 6	19:42902079-42919563	9.006492876	0.00038603	0.045389644
RPL7P3	ribosomal protein L7 pseudogene 3	20:62572671-62573384	6.502978498	0.00038737	0.045397412
KRTAP4-6	keratin associated protein 4-6	17:41139433-41140487	-6.42831147	0.00038899	0.04543759
AC113133.1	novel transcript, antisense to ANK1	8:41660991-41665566	NA	0.00039044	0.045457671
KRTAP4-2	keratin associated protein 4-2	17:41177446-41178208	-6.8229164	0.00039179	0.045466699
LCE3C	late cornified envelope 3C	1:152600662-152601086	-6.51016253	0.00039382	0.045553375
SULF2	sulfatase 2	20:47656348-47786616	2.177230046	0.00040695	0.046919797
CRYAB	crystallin alpha B	11:111908565-111923722	3.092053153	0.00041008	0.046977128
MATN3	matrilin 3	2:19992111-20012694	3.427426899	0.00040878	0.046977128
DWORF	DWARF open reading frame	3:155290229-155293775	5.749259566	0.00041147	0.046985875
ARX	aristaless related homeobox	X:25003694-25016420	6.932651414	0.00041292	0.047000701
LGALS13	galectin 13	19:39602501-39607476	NA	0.00042829	0.048298892
PLPP7	phospholipid phosphatase 7 (inactive)	9:131289694-131309262	3.059625269	0.0004284	0.048298892
AC046195.1	novel transcript	8:137809444-138083570	NA	0.00043308	0.048673504
FRMPD1	FERM and PDZ domain containing 1	9:37651000-37746904	-2.47218937	0.00044161	0.049320731
