# Supplementary material for: Four calcium signaling pathway-related genes were upregulated in microcystic adnexal carcinoma: transcriptome analysis and immunohistochemical validation
Source: World J Surg Oncol. 2022 May 4;20:142. doi: 10.1186/s12957-022-02601-6 (PMC9066904; doi:10.1186/s12957-022-02601-6)
Supplement: Supplementary file 5 — Additional file 5: Supplemental Table S4. Immunohistochemical and molecular markers that have been reported in MAC. [file 12957_2022_2601_MOESM5_ESM.pdf]

Supplemental Table S4. Immunohistochemical and molecular markers that have been reported in MAC.

Molecular markers	Type	Pattern	Literature
CEA	Protein	Ductal lining cells	Nickoloff BJ. et al. (1986)
CK	Protein	Diffuse positive	Miyamoto T. et al. (1990)
CK5/6	Protein	Diffuse positive	Zhang L. et al. (2020)
CK7	Protein	Diffuse positive	Kathleen J. et al. (2001)
CK15	Protein	Diffuse positive	Hoang MP. et al. (2008)
CK19	Protein	Diffuse positive	Sellheyer K. et al. (2013)
CK20	Protein	Negative	Kathleen J. et al. (2001)
EMA	Protein	Diffuse positive	Mark R. et al. (1990)
p53	Protein	Moderately intense staining	Kathleen J. et al. (2001)
Bcl-2	Protein	Focally positive	Kathleen J. et al. (2001)

Ki-67	Protein	Less than 5%	Kathleen J. et al. (2001)
c-erbB-2	Protein	Negative	Kathleen J. et al. (2001)
Ber-EP4	Protein	Negative	Krahl D. et al. (2007)/Sellheyer K. et al. (2013)
α-SMA	Protein	Diffuse positive	Kathleen J. et al. (2001)
CD34	Protein	Negative	Kathleen J. et al. (2001)
CD23	Protein	Ductal lining cells	Carvalho J. et al. (2007)
p63	Protein	Periphery of tumor nests and minimal staining in the center of tumor islands	Vidal CI. et al. (2010)
Adipophylin	Protein	Sebaceous foci	Fernandez-Flores A. et al. (2018)
Ln-ɤ 2	Protein	Diffuse positive	Koga K. et al. (2020)
p-STAT3	Protein (Gene)	Diffuse positive (*JAK1* mutant)	Chan MP. et al. (2020)
p53/ *TP53*	Protein (Gene)	Diffuse positive (*TP53* mutant)	Chan MP. et al. (2020)
TP53	Gene	Mutation	Chen MB. et al. (2017)
CDKN2A	Gene	Chromosomal losses	Chen MB. et al. (2017)
CDKN2B	Gene	Chromosomal losses	Chen MB. et al. (2017)
